# Transethmoidal encephalocele associated with granulomatosis with polyangiitis: A case report

**DOI:** 10.1016/j.radcr.2026.08.023

**Published:** 2026-09-05

**Authors:** Vivian Soraya Siabatto-Cleves, Johana Andrea Sisa-Rodríguez, Carlos Alfonso Cortés-Ortega, Jason Javier Sisa-Rodríguez

**Affiliations:** a Department of Diagnostic Imaging, Universidad Nacional de Colombia, Bogotá, Colombia; b Department of Diagnostic Imaging, Hospital Universitario Nacional de Colombia, Bogotá, Colombia

**Keywords:** Encephalocele, Granulomatosis with polyangiitis, Wegener granulomatosis, Skull base defect, Sinonasal disease, Seizures

## Abstract

Encephaloceles are uncommon lesions, most of which are congenital, and may be overlooked in routine radiologic practice. Failure to identify a skull base defect and characterize the herniated contents may delay recognition of associated abnormalities and clinically important complications, including cerebrospinal fluid leakage, intracranial infection, intracranial hypotension, hydrocephalus, and seizures. Granulomatosis with polyangiitis can cause destructive sinonasal disease with extension to the skull base; however, a meningoencephalocele in this setting is exceptionally rare. We report a 58-year-old woman with longstanding granulomatosis with polyangiitis who presented with new-onset seizures. Computed tomography and magnetic resonance imaging demonstrated a right transethmoidal meningoencephalocele through an ethmoidal roof defect contiguous with extensive destructive sinonasal changes, with encephalomalacia and gliosis of the herniated brain parenchyma.

## Introduction

**Fig. 1:**
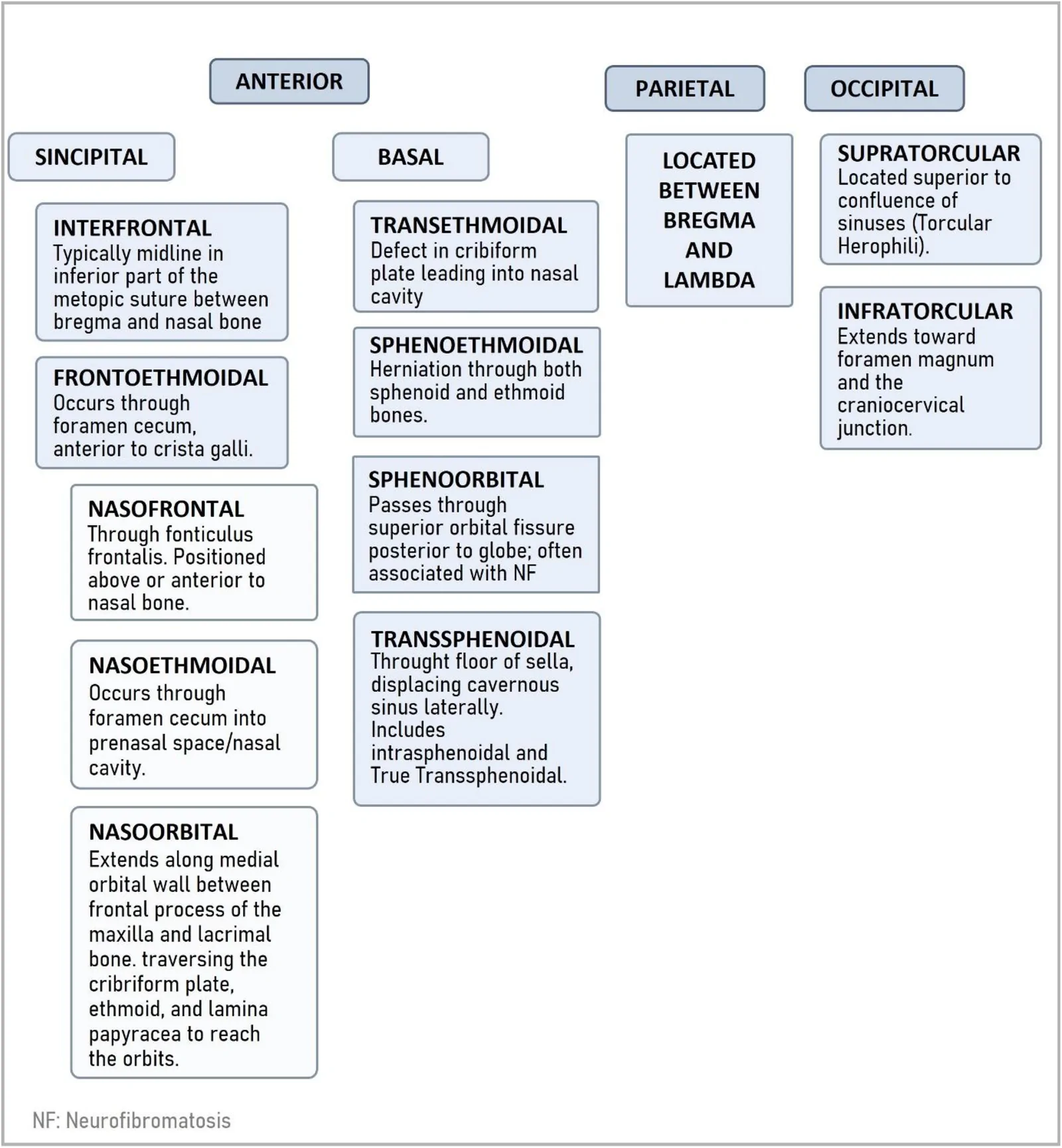
Anatomical classification of encephaloceles by location. Anterior encephaloceles are located anterior to the bregma and are subdivided into sincipital and basal types; parietal encephaloceles are located between the bregma and lambda; and occipital encephaloceles are located between the lambda and foramen magnum. The principal anatomical landmarks and subtypes are shown [5]. NF, neurofibromatosis.

Encephaloceles are defined by herniation of intracranial contents through a congenital or acquired skull defect. Congenital encephaloceles occur in approximately 1 in 4000 live births and account for 10%-20% of craniospinal dysraphisms, which collectively comprise nearly one-third of malformations identified during the perinatal period [[1], [2], [3], [4]]. Proposed mechanisms include abnormal neurulation, failed midline mesodermal migration, or defective neural tube closure secondary to incomplete fusion of the ossification centers [5]. Acquired encephaloceles may result from trauma, surgery, neoplasia, infection, inflammatory disease, vasculitis, spontaneous skull base defects, or cocaine-induced destructive sinonasal disease [4,5]. Based on anatomic location, encephaloceles are classified as occipital, parietal, or anterior; occipital lesions account for approximately 75% of congenital cases (Fig. 1) [1,4].

Granulomatosis with polyangiitis (GPA) is a systemic necrotizing granulomatous vasculitis affecting small- and medium-sized vessels and is associated with antineutrophil cytoplasmic antibodies (ANCA). Upper and lower respiratory tract involvement is common [6]. Sinonasal disease occurs in up to 70% of patients with GPA, affecting approximately one-third of patients with localized disease and 64%-84% of those with generalized disease [7,8]. Common clinical manifestations include chronic rhinosinusitis, serosanguineous nasal discharge, hyposmia, nasal obstruction, epistaxis, and facial pain [7]. Typical imaging findings include osseus erosion, reported in 59%-62% of patients; neo-osteogenesis, present in more than 50%; and nodular mucosal thickening, described on up to 87% of magnetic resonance imaging (MRI) examinations [[8], [9], [10]].

Advanced sinonasal GPA may cause extensive destruction of the nasal cavity and paranasal sinuses and, less commonly, extend to the anterior skull base. An acquired meningoencephalocele in this setting is exceptionally rare, with only one previously reported case [3]. We describe a right transethmoidal meningoencephalocele associated with extensive destructive sinonasal changes in a patient with longstanding GPA who presented with new-onset seizures.

## Case presentation

A 58-year-old woman with a 20-year history of GPA underwent outpatient brain MRI and computed tomography (CT) of the paranasal sinuses as part of the evaluation of new-onset seizures. GPA had been diagnosed by the rheumatology service in 2004 after a 1-year history of nonspecific ophthalmologic and otologic symptoms and a positive cytoplasmic antineutrophil cytoplasmic antibodies (c-ANCA). Cutaneous manifestations subsequently developed; however, skin biopsy was nondiagnostic, and the diagnosis was established on clinical and serologic grounds. At the time of imaging, the patient was receiving maintenance therapy with azathioprine and deflazacort; biologic therapy was considered but was not initiated because of recurrent infections. She denied prior sinonasal or cranial surgery, occupational silica exposure, cocaine use, and a family history of rheumatologic disease. She reported a remote history of occasional cigarette smoking for less than 6 months in her 20s.

**Fig. 2:**
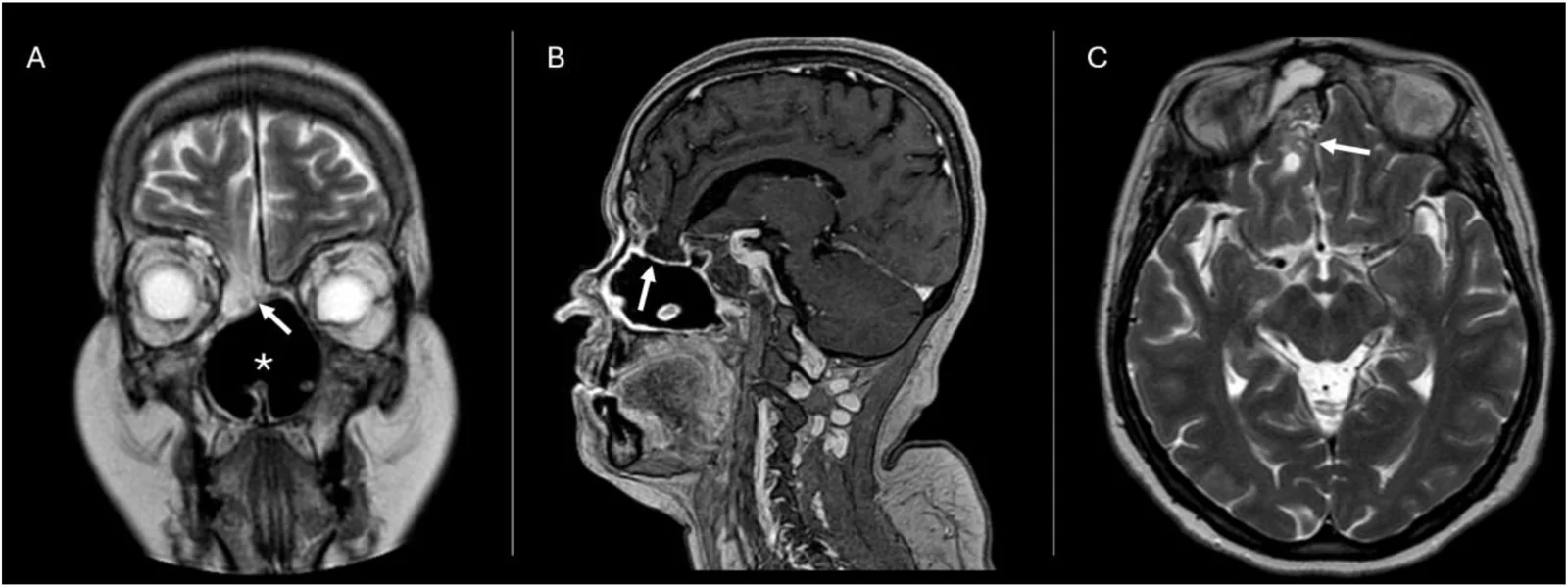
Brain magnetic resonance images. (A) Coronal T2-weighted image demonstrating extensive erosive involvement of the nasal cavity (*), with a right ethmoidal roof defect and herniation of the orbitofrontal gyri, which demonstrates T2 hyperintensity (arrow). (B) Contrast-enhanced sagittal T1-weighted image shows pachymeningeal enhancement within the hernia sac (arrow). (C) Axial T2-weighted image shows cortical thinning and adjacent white matter T2 hyperintensity consistent with encephalomalacia and gliosis.

CT and MRI demonstrated a right transethmoidal meningoencephalocele through a 20 × 16-mm defect in the ethmoidal roof, with herniation of the olfactory fossa contents and adjacent frontobasal brain parenchyma. The hernia sac measured 19 × 15 × 9 mm. The herniated parenchyma demonstrated encephalomalacia and gliosis, and contrast-enhanced MRI showed pachymeningeal enhancement within the sac (Fig. 2). No cerebrospinal fluid (CSF) leak was documented, and imaging showed no pneumocephalus or signs of intracranial hypotension.

**Fig. 3:**
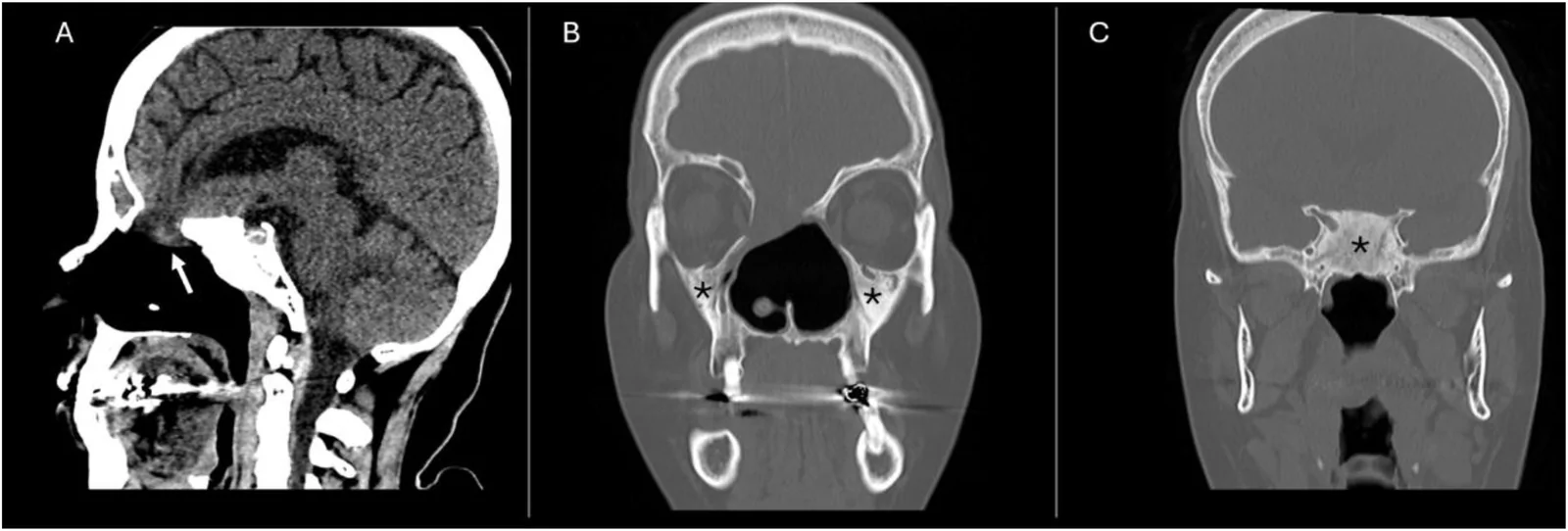
Computed tomography images of the paranasal sinuses. (A) Sagittal soft-tissue window image shows a right ethmoidal roof defect with herniation of brain parenchyma (arrow). (B) Coronal bone window image shows extensive osseous destruction of the nasal cavity and ethmoid air cells, with sclerotic changes in the maxillary sinuses (*). (C) Coronal bone window image shows sclerotic change in the sphenoid sinus (*).

CT of the paranasal sinuses demonstrated extensive osseous destruction of the sinonasal cavity, including complete loss of the ethmoid air cells, the bilateral superior, middle, and inferior turbinates, and the nasal septum. Additional sclerotic changes consistent with neo-osteogenesis involved the maxillary and sphenoid sinuses (Fig. 3). On the basis of these imaging findings, urgent neurosurgical and otorhinolaryngologic evaluation was recommended because the transethmoidal meningoencephalocele was considered a probable structural explanation for the seizures. Clinical records reviewed 21 months after image acquisition confirmed that conservative management had been pursued without surgical repair. The patient remained on maintenance therapy with azathioprine and deflazacort, received lacosamide for seizure control, and continued under multidisciplinary follow-up with Neurology, Otorhinolaryngology, and Rheumatology. No seizure recurrence was documented during the available follow-up period.

## Discussion

In this case, CT and MRI demonstrated a right ethmoidal roof defect with complete loss of the ethmoid air cells and herniation of the meninges and brain parenchyma, including the genu of the corpus callosum, right gyrus rectus, and right middle orbital gyrus, into the ethmoid sinus and anterior nasal cavity. The herniated brain parenchyma showed encephalomalacia and gliosis. Complete destruction of the ethmoid air cells and other nasal cavity structures, together with maxillary and sphenoid sinus sclerosis, reflected advanced chronic sinonasal disease. In the setting of longstanding GPA, the direct anatomic continuity between the sinonasal destruction and the anterior skull base defect favors, but does not establish, an acquired mechanism.

To date, only one other encephalocele has been reported in association with GPA [3]. Short et al. [3] described a 46-year-old man in whom a transalar meningoencephalocele was identified during preoperative evaluation of chronic sinonasal symptoms. By contrast, our patient underwent imaging for new-onset seizures, and the transethmoidal meningoencephalocele was considered a probable structural explanation. The imaging patterns also differed. The previously reported lesion extended through an approximately 3.2-cm defect in the left greater sphenoid wing centered at the expected location of the foramen ovale and contained inferior temporal lobe tissue. In our case, the lesion extended through a 20 × 16-mm defect in the right ethmoidal roof and contained frontobasal brain parenchyma with encephalomalacia and gliosis, in association with extensive destructive sinonasal changes. Both cases demonstrated pachymeningeal enhancement. The transalar lesion may have represented a developmental skull base defect with superimposed GPA-related meningeal inflammation, whereas the pattern in our patient favors, but does not prove, an acquired defect. Management also differed: the previously reported patient underwent endoscopic sinonasal surgery, with no encephalocele repair described, whereas our patient was managed conservatively with antiseizure therapy and had no documented seizure recurrence during the 21-month follow-up period.

Although GPA-associated meningoencephaloceles are exceptionally rare, radiologists should recognize and accurately characterize these lesions because the imaging findings directly inform referral, surgical planning, and follow-up. The imaging features emphasized by Pal et al. [5] provide a useful framework for structured assessment, including the anatomic location and dimensions of the skull defect and hernia sac; the sac contents; signal abnormalities within the herniated parenchyma; associated malformations; hydrocephalus; CSF leakage; pneumocephalus; intracranial hypotension; and evidence of superimposed infection. Although this framework was developed primarily for congenital encephaloceles, it is also applicable to acquired lesions. Both the dimensions of the skull defect and those of the hernia sac should be reported in 3 orthogonal planes, as giant encephaloceles defined by a hernia sac larger than the patient's head—are associated with a poorer prognosis [5]. Terminology should reflect the contents of the hernia sac. A meningocele contains meninges and CSF, whereas a meningoencephalocele also contains brain parenchyma, which is often gliotic. Atretic encephaloceles contain dura, fibrous tissue, and degenerated neural tissue, whereas glioceles are CSF-containing cysts lined by glial tissue [5]. In the present case, the presence of herniated brain parenchyma supports the term transethmoidal meningoencephalocele. In acquired lesions, imaging should also be scrutinized for a CSF fistula and complications such as pneumocephalus, intracranial hypotension, and infection. Leptomeningeal enhancement and subarachnoid fluid-attenuated inversion recovery (FLAIR) hyperintensity may suggest central nervous system infection and should be interpreted in the appropriate clinical context.

When extensive midline sinonasal destruction is encountered without an established diagnosis, the differential diagnosis includes sarcoidosis, relapsing polychondritis, immunoglobulin G4-related disease, invasive and chronic infections, extranodal natural killer/T-cell lymphoma, and cocaine-induced midline destructive lesions. Relevant infections include invasive fungal rhinosinusitis, rhinosporidiosis, rhinoscleroma, syphilis, tuberculosis, leprosy, and leishmaniasis. These entities may overlap clinically, radiologically, and histopathologically, requiring integration of exposure history, serologic testing, microbiologic studies, and tissue sampling when appropriate [11].

## Conclusion

A transethmoidal meningoencephalocele associated with destructive sinonasal GPA is exceptionally rare. CT is essential for defining osseous destruction and the skull base defect, whereas MRI characterizes the herniated contents, associated parenchymal injury, and meningeal enhancement. Recognition of this pattern should prompt assessment for CSF leakage and intracranial complications and timely multidisciplinary referral. The close anatomic relationship between the sinonasal destruction and the ethmoidal roof defect in this case favors, but does not establish, an acquired GPA-related mechanism. To our knowledge, this is the second reported meningoencephalocele associated with GPA and the first reported at a transethmoidal location.

## Authors contribution

Vivian Soraya Siabatto Cleves: Conceptualization, Investigation, Writing – original draft. Johana Andrea Sisa Rodríguez: Conceptualization, Investigation, Visualization, Supervision, Writing – original draft, Writing – review & editing. Carlos Alfonso Cortés Ortega: Conceptualization, Supervision, Writing – review & editing. Jason Javier Sisa Rodríguez: Conceptualization, Supervision, Writing – review & editing. All authors read and approved the final manuscript. All authors meet the authorship criteria according to the ICMJE guidelines.

## Patient consent

Written informed consent was obtained from the patient for publication of this case report and any accompanying images.
